# Palliative radiotherapy in addition to self-expanding metal stent for improving dysphagia and survival in advanced oesophageal cancer (ROCS: Radiotherapy after Oesophageal Cancer Stenting): study protocol for a randomized controlled trial

**DOI:** 10.1186/1745-6215-15-402

**Published:** 2014-10-22

**Authors:** Douglas Adamson, Jane Blazeby, Annmarie Nelson, Chris Hurt, Lisette Nixon, Jim Fitzgibbon, Tom Crosby, John Staffurth, Mim Evans, Noreen Hopewell Kelly, David Cohen, Gareth Griffiths, Anthony Byrne

**Affiliations:** Tayside Cancer Centre, Ward 32, Ninewells Hospital, Dundee, DD1 9SY UK; School of Social & Community Medicine, University of Bristol, Canynge Hall, 39 Whatley Road, Bristol, BS8 2PS UK; Marie Curie Palliative Care Research Centre, School of Medicine, College of Biomedical and Life Sciences, Cardiff University, 1st Floor, Neuadd Meirionnydd, Heath Park, Cardiff, CF14 4YS UK; Wales Cancer Trials Unit, School of Medicine, College of Biomedical and Life Sciences, Cardiff University, 6th Floor, Neuadd Meirionnydd, Heath Park, Cardiff, CF14 4YS UK; Velindre Cancer Centre, Velindre Hospital, Whitchurch, Cardiff, CF14 2TL UK; Institute of Cancer and Genetics, School of Medicine, College of Biomedical and Life Sciences, Cardiff University, Heath Park, Cardiff, CF14 4XW UK; National Institute for Social Care and Health Research (NISCHR), Clinical Research Centre, 3rd Floor 12 Cathedral Road, Cardiff, CF11 9LJ UK; NISCHR Welsh Health Economics Support Service, University of South Wales, Pontypridd, CF37 1DL UK; University of Southampton Clinical Trials Unit, MP 131, Southampton General Hospital, Tremona Road, Southampton, SO16 6YD UK

**Keywords:** Esophageal cancer, Dysphagia, Stent, Radiotherapy, Palliative, Patient experience

## Abstract

**Background:**

The single most distressing symptom for patients with advanced esophageal cancer is dysphagia. Amongst the more effective treatments for relief of dysphagia is insertion of a self-expanding metal stent (SEMS). It is possible that the addition of a palliative dose of external beam radiotherapy may prolong the relief of dysphagia and provide additional survival benefit. The ROCS trial will assess the effect of adding palliative radiotherapy after esophageal stent insertion.

**Methods/Design:**

The study is a randomized multicenter phase III trial, with an internal pilot phase, comparing stent alone versus stent plus palliative radiotherapy in patients with incurable esophageal cancer. Eligible participants are those with advanced esophageal cancer who are in need of stent insertion for primary management of dysphagia. Radiotherapy will be administered as 20 Gray (Gy) in five fractions over one week or 30 Gy in 10 fractions over two weeks, within four weeks of stent insertion. The internal pilot will assess rates and methods of recruitment; pre-agreed criteria will determine progression to the main trial. In total, 496 patients will be randomized in a 1:1 ratio with follow up until death. The primary outcome is time to progression of patient-reported dysphagia. Secondary outcomes include survival, toxicity, health resource utilization, and quality of life. An embedded qualitative study will explore the feasibility of patient recruitment by examining patients’ motivations for involvement and their experiences of consent and recruitment, including reasons for not consenting. It will also explore patients’ experiences of each trial arm.

**Discussion:**

The ROCS study will be a challenging trial studying palliation in patients with a poor prognosis. The internal pilot design will optimize methods for recruitment and data collection to ensure that the main trial is completed on time. As a pragmatic trial, study strengths include collection of all follow-up data in the usual place of care, and a focus on patient-reported, rather than disease-orientated, outcomes. Exploration of patient experience and health economic analyses will be integral to the assessment of benefit for patients and the NHS.

**Trial registration:**

The trial was registered with Current Controlled Trials (registration number: ISRCTN12376468) on 10 July 2012.

## Background

Esophageal cancer resulted in 7,606 deaths in the United Kingdom in 2008, reflecting a 70% increase in male age-standardized mortality rates compared to 1971. It is the sixth most common cause of cancer deaths (fourth in men) and incidence rates are increasing by 4.2% per annum [1]. Prognosis is poor, with five-year survival rates of 10 to 15% [2]. It is predominately a disease of the elderly, with prevalence highest in the seventh and eighth decades of life. Most patients present with incurable disease, and for advanced disease, mean survival is three to five months [3].

The emphasis of treatment for the majority of patients is therefore on effective palliative interventions, with 70 to 90% requiring intervention for dysphagia [4, 5]. This single symptom has profound impact on social and physical functioning and other aspects of quality of life (QoL). Interventions to improve swallowing must therefore aim to produce prompt and lasting palliation of dysphagia whilst minimizing the need for late re-interventions and hospitalization. Interventions must produce these benefits without causing significant impairment of other aspects of QoL.

The most recent Cochrane systematic review [6] of interventions for dysphagia in esophageal cancer confirms the efficacy of self-expanding metal stents (SEMS) in providing rapid initial relief of dysphagia, with fewer adverse effects and lower re-intervention rates than endoscopic ablative therapies.

A health technology assessment (HTA) [7] also highlights the efficacy of stent placement. However, delayed complications are common and result in later re-interventions. A pragmatic study as part of that assessment found that 35% of patients receiving stents required re-interventions [8]. Homs *et al.* [9] in a comparative study of brachytherapy described a hemorrhage rate of 13% in patients treated with a stent within a median of 123 days post-insertion. Conio *et al*. [10] in a randomized comparison of two stent types described tumor overgrowth in 19% within a median of 97 days post-insertion.

It is such late re-interventions and complications which account for the major proportion of dysphagia treatment costs [8], requiring travel to hospital and inpatient stays which also impair QoL. This is consistent with estimations that healthcare costs in general in the last year of life account for 20 to 30% of overall healthcare budgets [11].

Of the non-stent interventions, brachytherapy studies [9, 12] suggest this treatment gives longer dysphagia-free survival and more stable QoL compared to a stent. However, a recent survey by the Royal College of Radiologists (London, United Kingdom) showed that brachytherapy treatment for all tumor sites accounts for only 2.5% of all radiotherapy patients. There is little access to, or expertise in, this type of radiotherapy for esophageal cancer patients in the United Kingdom [13]. In contrast, external beam radiotherapy is readily accessible by patients at regional cancer centers across the United Kingdom, although its use in the immediate post-stent period has not been rigorously studied and is not routinely used.

Available evidence therefore suggests that a stent is an appropriate intervention for rapid dysphagia relief in incurable esophageal cancer. The efficacy of stent alone, however, is limited by early problems with pain, decline in general aspects of QoL, and later complications such as hemorrhage and tumor overgrowth. Re-interventions not only impose significant burden on NHS resources but decrease the QoL and functioning of an unwell, predominantly elderly, population. Combination with other treatments might reduce costs and patient burden; for example the addition of radiotherapy may ameliorate these problems and provide additional survival benefit.

Given the sporadic and consistently limited availability of brachytherapy in the United Kingdom, the overarching aim of this study is to address uncertainties in the current evidence base by assessing whether the addition of external beam radiotherapy prolongs improvement in dysphagia, improves QoL, and reduces health economic and personal burden in patients undergoing stent placement.

### Main research question

The main study aim is to assess the impact of palliative radiotherapy in addition to stent placement on the time to progression of patient-reported dysphagia in a patient population unable to undergo surgery or radical chemo-radiotherapy. We will also examine the impact on overall survival, serious adverse events (SAEs), toxicity, QoL, and cost effectiveness. The feasibility of patients’ recruitment to the trial will be examined in the internal pilot phase and the participants’ experience of the trial participation and interventions will be explored in the qualitative research study.

## Methods/Design

**Figure 1:**
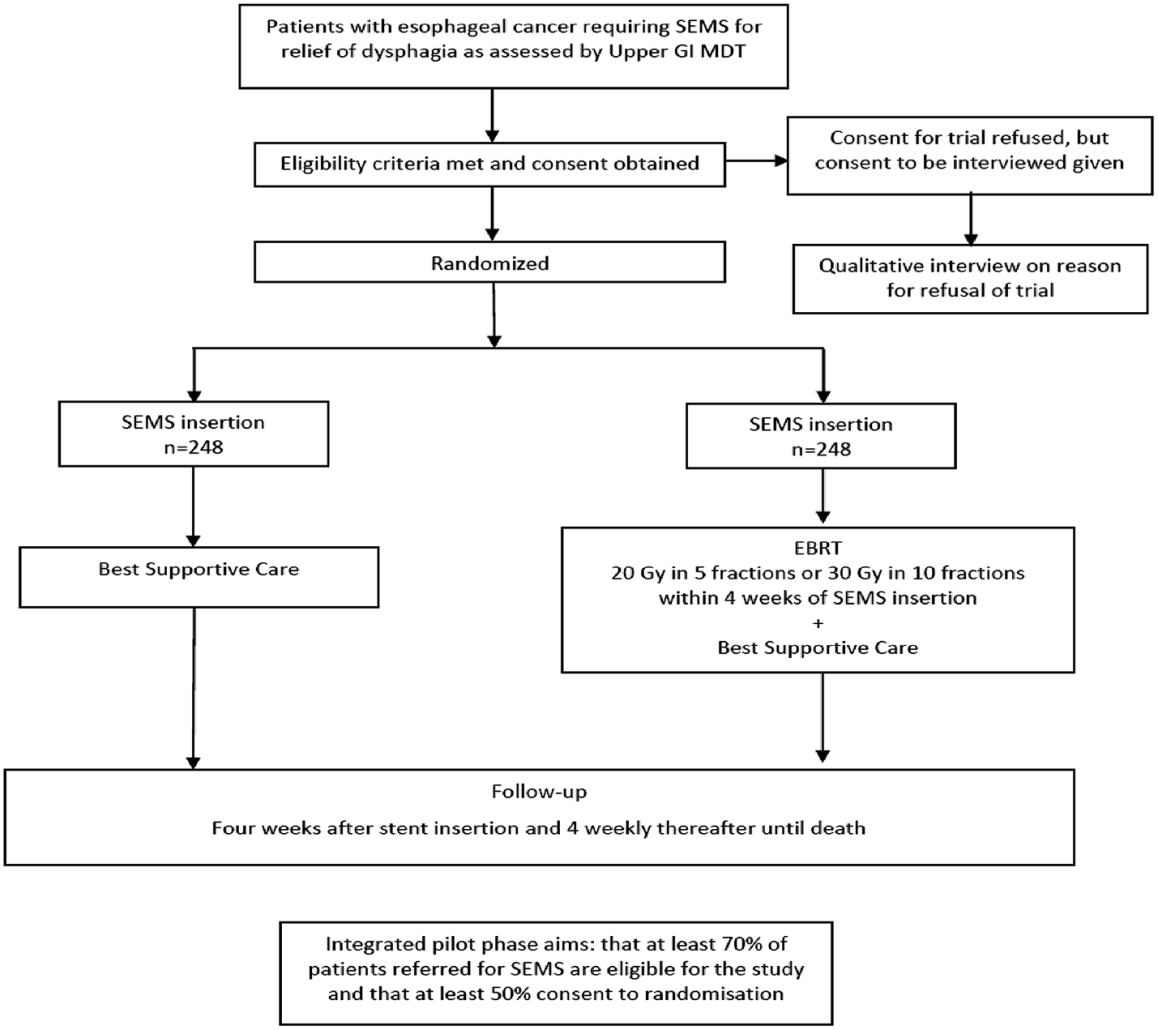
**Trial schema.** Integrated pilot phase aims: that at least 70% of patients referred for SEMS are eligible for the study and that at least 50% consent to randomization. SEMS: Self Expanding Metal Stent; GI MDT: Gastrointestinal MDT; EBRT: External Beam Radiotherapy; Gy: Gray.

This will be a pragmatic, randomized controlled trial, with an internal pilot phase, of external beam radiotherapy in addition to stent versus stent alone in patients clinically assessed as requiring stent insertion for the relief of dysphagia caused by esophageal cancer (Figure 1). In compliance with the Helsinki declaration, the Radiotherapy after Oesophageal Cancer Stenting (ROCS) study has been granted ethical approval by the Southeast Wales Research Ethics Committee (reference number: 12/WA/0230).

The target population will be patients selected by an upper gastrointestinal multidisciplinary team (MDT) for palliation of malignant dysphagia with an esophageal stent. Patients will be identified in secondary care, including cancer centers and district general hospitals, from 10 participating centers. All study centers have been chosen on the basis of number of patients supported by the MDT and potential recruitment rates, as well as interest in the study. Geographical spread has also been an important consideration, in recognition of higher incidence rates of esophageal cancer in particular parts of the United Kingdom including Scotland, North Wales, and Northwest England.

### Internal pilot

This is a pragmatic study in a patient group who are likely to be frail and elderly. To ensure that study recruitment is feasible, and that study conduct is optimized, the trial incorporates an internal pilot phase. The study will be limited to five centers in the first year, with continuation of the study dependent on demonstration of feasibility as defined by the following rules agreed by discussion within the trial management group: (1) at least 70% of patients undergoing stent placement as primary treatment for esophageal cancer dysphagia are considered eligible for the trial (if necessary, by revising the eligibility criteria); and (2) at least 50% of eligible patients consent to randomization in the first nine months of recruitment.

A screening log will record the details of patients who are or are not screened in full for trial entry, and the precise reasons for ineligibility. The screening log will also record details of eligible participants who do not consent to randomization. The log will be used to understand barriers to trial recruitment and patient preferences. Anonymized data will be returned centrally on a monthly basis for review. A lead research practitioner will be part of the trial team to support research nurses at other sites and organize national meetings to review screening data, share best practice, and where appropriate, make recommendations for change. Following review, centers may be contacted as appropriate if potentially eligible patients are not being fully screened, or if many patients are being classified as ineligible. Site visits by the lead nurse will consider these details and discuss with the site team as necessary to ensure that recruitment of all patients potentially eligible for the trial is maintained. Qualitative assessments to understand patient experience of the recruitment process, focusing on non-consenting patients in the pilot phase, will further inform study conduct.

### Participant eligibility

Patients meeting the following nine criteria may be included in the trial: (1) have histological confirmation of esophageal carcinoma excluding small-cell carcinoma histology, (2) are not suitable for radical treatment (esophagectomy or radical chemo-radiotherapy) either because of patient choice or medical reasons, (3) have dysphagia clinically assessed as needing a stent as primary treatment of the dysphagia, (4) be aged 16 years or over, (5) discussion and treatment decision for stent (SEMS) placement made by an upper gastrointestinal multi-disciplinary team, (6) ability to attend for radiotherapy as determined by clinician assessment, (7) an expected survival time of at least 12 weeks, (8) willing to provide written informed consent, and (9) has completed baseline QoL questionnaires (as a minimum patients must have completed the Oesophago-Gastric 25 (OG25)questionnaire).

Patients meeting any of the following eight exclusion criteria will be excluded from the study: (1) have histology of small-cell carcinoma type; (2) have a tumor length of greater than 12 cm; (3) have tumor growth within 2 cm of the upper esophageal sphincter; (4) have endoscopic treatment of the tumor, other than dilatation, planned during the peri-stent period; (5) have a tracheo-esophageal fistula; (6) have a pacemaker in the proposed radiotherapy field; (7) have undergone previous radiotherapy in the area of the proposed radiotherapy field; (8) or are a female patient who is pregnant.

### Recruitment and randomization

Eligible participants will be identified and approached in secondary care including cancer centers and district general hospitals. The patient’s consent to participate in the trial will be obtained prior to any trial-related procedures, which includes the insertion of a stent, and after a full explanation of the treatment options has been given. Consent will be taken by an appropriately trained research nurse or delegate. Patients who consent to randomization will also be asked to consent to NHS Information Centre flagging so that the date and cause of death can be collected without longer term follow-up. This will be optional and additional to the standard informed consent.

Patients who decline to participate in the main study, as well as participants who enter the main study, will be asked whether they consent to the storage of their contact details so that a qualitative researcher may contact them to invite them to participate in a qualitative interview about the reasons behind their decision not to participate in the main trial, or of their experiences of the interventions, as appropriate. Patients who agree to the storage of their contact details will be asked to sign the appropriate section of the main consent form. At this time, they will also be given a separate qualitative interview patient information sheet and consent form to take home and read. The qualitative researcher will collect consent for conducting the interview immediately prior to commencement of the interview, using the qualitative interview consent form.

Patients will be randomized centrally by the Wales Cancer Trials Unit prior to stent insertion using the method of minimization, which includes a random element. Patients will be stratified for a number of clinically important factors. The randomization allocation ratio for control to intervention arms will be 1:1 and the embedded qualitative component of the study will also explore patients’ perceptions and experiences of the randomization process.

### Study interventions

The SEMS procedure for both arms will be as follows: a stent will be inserted, following the decision by the MDT to proceed with stenting as the primary treatment for the dysphagia. Insertion will be in accordance with the standard procedures of the treating centre. The length and type will be determined by the responsible clinician. The following will be recorded: whether the stent is inserted under sedation or general anaesthetic, whether dilatation is required before or after stent insertion, and whether radiological imaging is used. Where possible the length of stent will be chosen to ensure that at least 2 cm of normal esophagus is covered by the stent above and below the tumor stricture. Where necessary, more than one stent may be deployed. A post-insertion esophagogram may be used to confirm stent position and exclude perforation.

Esophageal dilatation that is used as part of the center’s normal procedure for stent insertion is permitted. The trial should not be offered to patients who are deemed to need or are offered routine endoscopic treatment of the tumor (such as endoscopic laser therapy) in the immediate peri-stenting period, unless an emergency situation arises that requires such a procedure. Brachytherapy or external beam radiotherapy should not be planned to be given routinely after stent insertion for those patients in the control arm. Patients will be stratified according to the prior use of systemic chemotherapy.

External beam radiotherapy treatment for the intervention arm will begin within four weeks after stent insertion. Radiotherapy will be delivered using a simple technique (usually a parallel opposed pair of beams) with either a dose of 20 Gy in five fractions over one week, or 30 Gy in 10 fractions over two weeks, prescribed to the mid-point of the irradiated volume, using daily fractionation and the center’s normal radiotherapy treatment procedures. The dose and fractionation schedule chosen will be at the discretion of the treating clinical oncologist. Treatment will normally be received as an outpatient.

If the patient misses more than seven consecutive calendar days during radiotherapy treatment, then they should be withdrawn from the intervention and further treatment given at the clinician’s discretion. In the unlikely event of radiotherapy side effects severe enough to interfere with treatment delivery, the treating clinician may temporarily stop treatment and allow a break of no more than seven calendar days prior to recommencement.

The Radiotherapy Quality Assurance (RTQA) will be carried out by the Cardiff National Cancer Research Institute (NCRI) Radiotherapy Trials Quality Assurance (RTTQA) Group. RTQA accreditation is required by all centers, but due to the simple nature of the radiotherapy delivered in this trial, will not be extensive and will consist of pre-trial and on-trial quality assurance.

#### Pre-trial quality assurance

A process document is to be completed by the radiotherapy site prior to being opened to recruitment. This should contain information on set-up, verification, and beam arrangement. This will be reviewed by the ROCS RTQA group. The national radiotherapy trials quality assurance baseline questionnaire should be returned to the NCRI RTTQA group, if not updated within the last two years.

#### On-trial quality assurance

Following entry of the first patient into the trial at a radiotherapy treatment site, a set of CT images or simulator images, together with information concerning the treatment fields (Digital Imaging and Communications in Medicine-RadioTherapy file or hard copy) and treated volumes should be forwarded to the ROCS RTQA group.

### Assessments and outcomes

A research practitioner will collect baseline data prior to stent insertion, one week following stent insertion, four weeks post stent insertion (and after radiotherapy in the intervention arm) and four-weekly thereafter until death. Dedicated research staff will visit patients at home or a place of their choice and the patients will be asked to complete the questionnaires themselves whenever possible.

The assessments at baseline will be measured using WHO performance status, QoL questionnaires (European Organisation for Research and Treatment of Cancer Quality of Life Questionnaire C30(EORTC QLQ-C30), EORTC QLQ-OG25, EuroQuol-5D (EQ-5D)) and the toxicity assessment Common terminology Criteria for Adverse Events (CTCAE) v4.03.

The assessment performed within one week of stent insertion will be measured using WHO performance status, questionnaires (EORTC QLQ-C30, EORTC QLQ-OG25, EQ-5D), the toxicity assessment (CTCAE v4.03), stent morbidity data, and a qualitative interview (subset of patients).

The assessments performed four weeks after stent insertion and four-weekly thereafter until death will be measured using the WHO performance status, questionnaires (EORTC QLQ-C30, EORTC QLQ-OG25, EQ-5D), the toxicity assessment (CTCAE v4.03), stent morbidity data, qualitative interview (subset of participants, week four and week eight only).

### Primary outcome measure

#### Patient-reported dysphagia

This will be measured at the specified time points using the EORTC QLQ-OG25 questionnaire. The QLQ-OG25 is an updated and improved questionnaire [14] that amalgamates the widely-used EORTC scales to assess health-related quality of life (HRQoL) in patients with esophageal and gastric cancer [15, 16]. Dysphagia has been chosen because it is the dominant symptom in advanced esophageal cancer and the aim of treatment is to alleviate this problem effectively and maintain effective swallowing for as long as possible. The new questionnaire has six scales and the dysphagia scale is scored from 0 to 100; a change of 10 to 15 points is considered clinically significant [17].

Relief of dysphagia is expected in the majority of participants following stent insertion. A dysphagia score will be taken at baseline (prior to the stent insertion) and then one week after stent insertion (prior to radiotherapy in the intervention arm). This second measurement will form the ‘time zero’ measurement for the main endpoint of the study. If no improvement from baseline of more than 11 points is observed at the ‘time zero’ measurement, then patients will remain in the study but will be documented as a failure at ‘time zero’ and will undergo further interventions undertaken at the discretion of the treating physician. All patients will then be followed up on at four weeks after stent insertion, and at four-weekly intervals after that. The time point at which an 11-point deterioration is seen in the dysphagia scores compared to the ‘time zero’ measurement will form the event for the primary outcome. Following this time point (progression in dysphagia), patients will continue to be followed up on four-weekly until death. It is possible that patients undergoing radiotherapy may have a temporary worsening of dysphagia secondary to radiation-induced esophagitis, and other temporary changes might occur. This will be important to capture. However, to ensure that it does not bias the primary outcome, definitive deterioration in dysphagia will be defined as an 11-point change on two consecutive occasions, with the first being taken as the event time point. If there is missing data at that subsequent assessment, deterioration will be assumed and timed at the previous assessment.

### Secondary outcome measures

#### Quality of life

QoL will be measured using the EORTC QLQ-C30, EORTC QLQ-OG25, and EQ-5D at the time points described. The EORTC QLQ-C30 has become a benchmark measure of QoL in cancer patients. This measure will be employed in addition to QLQ-OG25 as validation of the latter. Lagergren *et al*. [14] demonstrated that they measure separate HRQoL issues, and it is likely that dysphagia only accounts for a proportion of QoL impact [18]. The EQ-5D [19] is a short QoL tool which is designed to complement other QoL measures, and is recommended by the National Institute for Health and Care Excellence (NICE) for use in providing an index of HRQoL for generation of economic analyses.

#### Survival

Notification of death will be collected and overall survival will be calculated from the date of randomization to the date of death from any cause. Participants remaining alive will be censored at the date of last follow-up.

#### Morbidity

Overall length of hospital stay, complication rates, number of blood transfusions, and re-intervention rates will be gathered from case notes and captured in the case report forms. Early complications will be defined as those occurring within seven days of the intervention, and late complications will be defined as those occurring more than seven days after the intervention. Standard definitions of stent complications will be clearly described in the protocol.

Toxicity data will be scored using the National Cancer Institute CTCAE v4.03 and the Radiation Therapy Oncology Group acute/late questionnaire at baseline, during treatment, and at the pre-specified time points on follow-up. SAEs will be monitored in real-time by the chief investigator and trial management group (TMG.) Data will also be collected on symptom burden including pain, eating restrictions, and physical functioning via the EORTC QLQ-C30 and QLQ-OG25 scales.

#### Cost effectiveness

The economic evaluation will be in the form of a cost utility analysis assessing total costs against differences in HRQoL. This is the form of economic evaluation preferred by the NICE [20]. In line with NICE guidance, the analysis will be undertaken from an NHS and Personal Social Services perspective.

HRQoL will be assessed using EQ-5D which is a single index utility-based measure [21]. Quality-adjusted life years (QALYs) will be derived from the group differences in EQ-5D scores between baseline (after adjusting for any baseline differences [22]) and death using the area under the curve method. EQ-5D has been used previously to study patients with inoperable esophageal cancer [8].

Costs associated with stent insertion are not relevant to the study question. The only direct cost of the intervention is that associated with the delivery of radiotherapy which will be delivered according to centre protocols. The primary source of unit costs for radiotherapy will be the relevant NHS tariffs [23].

Data on total NHS resource use will be collected prospectively by the research nurses at baseline and at all time points specified above, via a combination of case note review and patient recall, and valued using relevant unit costs. Due to likely skewness, cost data will be bootstrapped [24]. Patient-borne costs, including travel to receive radiotherapy, will be monitored but will not be included in the cost utility analysis.

Cost effectiveness will be reported in the form of an incremental cost-effectiveness ratio. Uncertainty around individual parameters will be explored through a series of one-way sensitivity analyses, particularly with respect to the unit cost of radiotherapy, which will be examined through alternative costing methodologies [25]. Joint uncertainty will be explored through a probabilistic sensitivity analysis which will produce a cost-effectiveness acceptability curve showing the probability of the intervention being cost effective over a range of willingness-to-pay thresholds, such as the £30,000 per additional QALY currently used by NICE. As no long-term costs or benefits are anticipated given the short life expectancy of study patients, discounting will not be applied.

#### Qualitative study

Few studies have used qualitative methods to explore patients’ experiences of esophageal cancer [7]. Only one recent qualitative paper is available that focuses on dysphagia in patients with esophageal cancer [26] and no qualitative papers on stent placements in esophageal cancer patients could be traced. Analysis of available literature demonstrates patients shared concerns around issues of coping. How these were constructed and relayed differed by narrative, but the thematic finding demonstrates the value of researching patients’ experiences as an outcome in itself. Drawing on the experiences of patients, the ROCS qualitative study will focus on the following objectives: exploring patients’ motivation for participating in the trial, exploring reasons for refusing the trial, assessing patient experience and perceptions of each arm of the trial, providing patient outcome data in order to assess the feasibility of the trial design, and identifying potential improvements to the recruitment process. It is essential that the qualitative study takes place in the pilot phase of the trial in order to address challenges to recruitment prior to the continuation of the main trial.

Subsequent to the consent process the interviews will be conducted at either the participant’s home or an alternative quiet location. If a face to face interview is not possible, telephone or video-linked interviews are allowed where appropriate. It is anticipated that the interview will take between 30 minutes and one hour, however it may be stopped earlier if a patient appears fatigued or becomes unwell.

The qualitative component of ROCS aims to recruit a sample size of 12 to 20 patients for the experimental arm of the trial, and 6 to 10 patients for the control arm, with 6 to 10 non-consenting patients expected to consent to interview on reasons for not participating in the main trial. Non-consenters to the clinical trial will be interviewed on one occasion whereas those participating in the clinical trial will be interviewed three times; in weeks one and four to capture initial decision-making thoughts, and again at week eight to explore experiences of the intervention process and perceptions of any benefits or negatives experienced thus far.

Analysis of the qualitative interviews will be undertaken using an Interpretative Phenomenological Analysis (IPA) framework [27, 28]. IPA seeks to explain the meaning that events have for participants based on their own, everyday social reality. Each interview will initially be analyzed in isolation and focus on exploring how participants have interpreted their experiences. The analysis will be undertaken using a coding framework that will be developed alongside the search for emerging themes that are common across the transcripts.

The results of the analysis will be discussed and presented with the use of narrative extracts to support and illustrate claims and findings of the analysis. It is intended that the TMG will analyze the results to assess any recruitment challenges that may have been identified, and respond with potential alterations to the trial design. The findings of the qualitative study will also, where appropriate, be used to complement the full trial report.

### Sample size considerations

In a population with a median survival of approximately four months, an increase in median time to deterioration in self-reported dysphagia of four weeks is considered clinically meaningful. This is based on previous results in Homs *et al*. [9] and expert multidisciplinary and service user opinion. A survey of participating centers demonstrated clinician accord with this. Time to event will be calculated from the time of stent insertion to the time of deterioration (of 11 points or more) or death. Sample size is therefore based on a time to event analysis, to detect an increase in median time to deterioration in self-reported dysphagia of four weeks: from 12 to 16 weeks (equivalent to a hazard ratio (HR) of 0.75 and a difference in 12-week event rate of 50 versus 60%). Recruitment time will be 4.25 years with a six-month follow-up after the final patient is recruited. For 80% power with an alpha of 0.05 based on a two-sided log rank test, 198 patients per arm will be required, with 396 in total, which is a total of 384 events. Assuming 20% attrition, a total of 496 patients will be required. The degree of attrition is set at this higher level because of the vulnerability of the patient population.

### Statistical analysis

All analyses will be performed on a full intention-to-treat basis, that is, all patients randomized will be included, and all patients will be analyzed according to their allocated group whatever treatment they receive. Descriptive statistics of the patient characteristics within each treatment group will be presented (including a summary of the type of the treatment each patient actual received).

The main analysis will compare the time until self-reported dysphagia progression between the two groups using Kaplan-Meier curves and the log-rank test. The final analysis will take place when 384 events (deterioration or deaths) have been reported, which is expected to be approximately six months after accrual closes. Kaplan-Meier curves and log-rank tests will also be used to compare the two groups on the secondary outcomes of overall survival. It is possible the data for the secondary endpoints (such as toxicity) may be mature enough for analysis and presentation before the main overall survival primary endpoint. The secondary outcomes of proportions of morbidities and re-intervention rates will be compared using a chi-square test. The area under the curve of HRQoL scores will be compared using t-tests adjusted for follow-up interval and survival. The main analysis will be carried out when the required number of events has been reached (see proposed sample size above).

No formal subgroup analyses are planned to look at differences in primary and secondary endpoints between treatment groups within specific groupings based on patient characteristics. However exploratory analysis will be conducted to explore whether there is any consistent benefit from using radiotherapy after a stent in different subgroups by creating HR plots and carrying out tests for interaction or trend based on chi-square analysis. The statistical package STATA (StataCorp LP, Texas, USA) will be used for all analysis and a statistical analysis plan will be written before the data is analyzed.

## Discussion

This is a challenging study of palliation in a frail patient population with a limited prognosis. In view of the vulnerability of the patient group and the need to maximize data capture, this study will be conducted in a small number of centers (10) over a longer period of time. This will allow dedicated research staff to promote the study adequately in each locality, maximizing screening and recruitment and following patient progress into the home setting - supporting patients in completion of study measurements and avoiding extra hospital visits. The internal pilot design in five centers during the first year will allow assessment of screening and recruitment processes and the success of data capture prior to continuation to the full trial.

The HTA technology assessment of 2005 [7] conducted a literature review which found that only 0.55% of papers published since 1966 on esophageal cancer considered QoL issues, despite the inherently palliative nature of treatment options for the majority of patients. This current study focuses on QoL and patient experience as key outcomes. The qualitative aspect of the study is integral to its overall design, and is essential in exploring both the feasibility of patient recruitment, and the experience and perceived acceptability of the interventions. This mixed methodological approach will enhance assessment of the overall benefit for patients and the NHS of adding palliative radiotherapy to stent placement in the context of advanced esophageal cancer.

### Trial status

The ROCS trial opened to recruitment in December 2013, with a planned recruitment period of 4.25 years.

## Abbreviations

CTCAE: Common Terminology Criteria for Adverse Events
EORTC: European Organization for Research and Treatment of Cancer
EQ: EuroQol
GY: Gray
HR: Hazards Ratio
HRQoL: Health-Related Quality of Life
HTA: Health Technology Assessment
IPA: Interpretative Phenomenological Analysis
MDT: Multidisciplinary team
NCRI: National Cancer Research Institute
NICE: National Institute for Health and care Excellence
QALY: Quality-Adjusted Life Years
QoL: Quality of Life
RTTQA: Radiotherapy Trials Quality Assurance
RTQA: Radiotherapy Quality Assurance
SAE: Serious Adverse Effects
SEMS: Self-Expanding Metal Stent
TMG: Trial Management Group
WHO: World Health Organization.

## References

[1] **Cancer Research UK: Esophageal Cancer Mortality Statistics** [http://info.cancerresearchuk.org/cancerstats/types/oesophagus/mortality]

[2] Sundelof M, Ye W, Dickman PW, Lagergren A (2002). Improved survival in both histological subtypes of esophageal cancer in Sweden. Int J Cancer.

[3] O’Hanlon DM, Callanan K, Karat D, Crisp W, Griffin SM (1997). Outcome, survival, and costs in patients undergoing intubation for carcinoma of the esophagus. Am J Surg.

[4] Watkinson AF, Ellul J, Entwistle K, Mason RC, Adam A (1995). Esophageal carcinoma: initial results of palliative treatment with covered self expanding endoprostheses. Radiology.

[5] Scottish Audit of Gastric and Esophageal Cancer (2002). Report 1997–2000: A Prospective Audit.

[6] Sreedharan A, Harris K, Crellin A, Forman D, Everett SM (2009). Interventions for dysphagia in esophageal cancer. Cochrane Database Syst Rev.

[7] Shenfine A, McNamee P, Steen N, Bond J, Griffin SM (2005). A pragmatic, randomized controlled trial of the cost effectiveness of palliative therapies for patients with inoperable esophageal cancer. Health Technol Assess.

[8] Shenfine J, McName P, Steen N, Bond J, Griffin SM (2009). A randomized controlled clinical trial of palliative therapies for patients with inoperable esophageal cancer. Am J Gastroenterol.

[9] Homs MYV, Steyerberg EW, Eijkenboom WMA, Tilanus HW, Stalpers LJA, Bartelsman JFWM, van Lanschat JJB, Wijrdeman HK, Mulder CJJ, Reinders JG, Boot H, Aleman BMP, Kuipers EJ, Siersema PD, for the Dutch SIREC study group (2004). Single-dose brachytherapy versus metal stent placement for the palliation of dysphagia from esophageal cancer: multicenter randomized trial. Lancet.

[10] Conio M, Repici A, Battaglia G, De Pretis G, Ghezzo L, Bittinger M, Messmann H, Demarquay JF, Blanchi Togni M, Conigliaro R, Filiberti R (2007). A randomized prospective comparison of self-expandable metal stents in the palliation of malignant esophageal dysphagia. Am J Gastroenterol.

[11] Hoover DR, Crystal S, Kumar R, Sambamoorthi U, Cantor JC (2002). Medical expenditures during the last year of life: findings from the 1992–1996 Medicare current beneficiary survey. Health Serv Res.

[12] Bergquist H, Wenger V, Johnsson E, Nyman J, Ejnell H, Hammerlid E, Lundell L, Ruth M (2005). Stent insertion or endoluminal brachytherapy as palliation of patients with advanced cancer of the esophagus or gastro-esophageal junction: results of a randomized controlled clinical trial. Dis Esophagus.

[13] Royal College of Radiologists (2007). The Role and Development of Brachytherapy Services in the United Kingdom.

[14] Lagergren P, Fayers P, Conroy T, Steinf HJ, Sezerg O, Hardwick R, Hammerlid E, Bottomley A, Van Cutsemk E, Blazeby JM, on behalf of the European Organisation for Research Treatment of Cancer Gastrointestinal and Quality of Life Groups (2007). Clinical and psychometric validation of a questionnaire module, the EORTC QLQ-OG25, to assess health-related quality of life in patients with cancer of the esophagus, the oesophago-gastric junction and the stomach. Eur J Cancer.

[15] Blazeby JM, Conroy T, Hammerlid E, Fayers P, Sezer O, Koller M, Arraras J, Bottomley A, Vickery CW, Etienne PL, Alderson D (2003). Clinical and psychometric validation of an EORTC questionnaire module, the EORTC QLQ-OES18, to assess quality of life in patients with esophageal cancer. Eur J Cancer.

[16] Blazeby JM, Conroy T, Bottomley A, Vickery C, Arraras J, Sezer O, Moore J, Koller M, Turhal NS, Stuart R, Van Cutsem E, D'haese S, Coens C (2004). Clinical and psychometric validation of a questionnaire module, the EORTC QLQ-STO 22, to assess quality of life in patients with gastric cancer. Eur J Cancer.

[17] Cocks K, King MT, Velikova G, Martyn St-James M, Fayers PM, Brown JM (2011). Evidence-based guidelines for the determination of sample size and interpretation of the European Organization for the Research and Treatment of Cancer. Quality of Life Questionnaire Core 30. J Clin Oncol.

[18] Blazeby MJ, Williams MH, Brookes ST, Alderson D, Farndon JR (1995). Quality of life measurement in patients with esophageal cancer. Gut.

[19] **What is EQ-5D™** [http://www.euroqol.org]

[20] National Institute for Health and Clinical Excellence (2009). Methods for Technology Appraisals.

[21] EuroQoL (1990). A new facility for the measurement of health-related quality of life. Health Policy.

[22] Manca A, Hawkins N, Sculpher MJ (2005). Estimating mean QALYs in trial-based cost-effectiveness analysis: the importance of controlling for baseline utility. Health Econ.

[23] Department of Health (2010). Payment by Results: Chemotherapy and Radiotherapy - A Simple Guide.

[24] Effron B, Tibshirani R (1993). An Introduction to the Bootstrap.

[25] Norlund A (2003). Cost of radiotherapy. Acta Oncol.

[26] Wainright D, Donovan JL, Kavadas V, Cramer H, Blazeby JM (2007). Remapping the body: learning to eat again after surgery for esophageal cancer. Qual Health Res.

[27] Smith JA, Osborn M, Smith JA (2008). Interpretative Phenomenological Analysis. Qualitative Psychology: A Practical Guide to Methods.

[28] Smith JA, Eatough V, Lyons E, Coyle A (2008). Interpretative Phenomenological Analysis. Analysing Qualitative Data in Psychology.

